# Quantitative Analysis of the Effect of an Ectopic Beat on the Heart Rate Variability in the Resting Condition

**DOI:** 10.3389/fphys.2018.00922

**Published:** 2018-07-12

**Authors:** Ahyoung Choi, Hangsik Shin

**Affiliations:** ^1^Department of Software, Gachon University, Seongnam, South Korea; ^2^Department of Biomedical Engineering, Chonnam National University, Yeosu, South Korea

**Keywords:** ectopic effect, ectopic beat, ectopic interpolation, heart rate variability, interbeat interval

## Abstract

The purpose of this study is to quantitatively analyze the effect of an ectopic beat on heart rate variability (HRV) in the time domain, frequency domain, and in a non-linear analysis. A quantitative analysis was carried out by generating artificial ectopic beats that probabilistically contained a missed beat or a false-detected beat, and the statistical significance was evaluated though a comparison with an ectopic-free HRV by increasing the ratio of the ectopic beat in 0.1% increments from 0 to 50%. The effect of the interpolation on the ectopic HRV was also investigated by applying nearest-neighbor interpolation, linear interpolation, and cubic spline interpolation. The results confirmed a statistically significant difference (*P* < 0.05) even in the less-than-1% ectopic HRV in every domain. When interpolation was applied, there were differences according to the interpolation method used, but statistical significance was secured for an ectopic beat ratio from 1 to 2% to several tens of a percent. In the effect, linear interpolation, and spline interpolation were confirmed to have a higher effect on the high-frequency related HRV variables, and nearest-neighbor interpolation had a higher effect on low-frequency related variables.

## Introduction

Heart rate variability (HRV) refers to the mathematical analysis of changes in the interbeat interval (IBI) of the heart, and this technique can be used to back track autonomic nervous system (ANS) activity (Taskforce, 1996). An ectopic beat has a beat-to-beat interval that deviates from the normal heartbeat interval including unwanted additional beats or skipped beats. The major cause of ectopic beating is a problem in the cardiac conduction system expressed as premature atrial contractions or premature ventricular contractions. The ectopic beat can result in a beating interval that is too short or too long, so the IBI can become too large or too small. Since these ectopic beats are caused by abnormalities in cardiac conduction rather than as the effects of the autonomic nervous system, the results for HRV in assessments of ANS can be distorted if the heart beat variability is analyzed without removing ectopic beats (Taskforce, 1996).

In practice, false QRS detection is the biggest obstacle to an accurate HRV analysis. When calculating the interval between beats, a false QRS detection appears as an ectopic beat and causes a main error in the HRV analysis of a normal person without an arrhythmia. A failure in the QRS detection can have many causes, including the use of electrosurgical instruments, powerline interference, respiration effects, electromyogram (EMG) artifacts, loose contact of electrocardiogram (ECG) electrodes and motion artifacts (Friesen et al., 1990).

Previous studies have developed QRS detection algorithms, several of which achieve more than 99% accuracy (Kim and Shin, 2016). However, the technology for high-accuracy detection in an electrocardiogram with motion noise has not yet been clearly described. In other words, the occurrence of an ectopic beat is inevitable in actual ECG utilization.

Recently, the use of digital health care has proliferated, increasing the need for ambulatory ECG measurements. In addition to clinical uses, studies on ECG use in daily life to manage health, exercise, stress, and emotional well-being have been actively conducted (Taelman et al., 2009; Hynynen et al., 2010; Kaikkonen et al., 2010, 2012; Choi et al., 2012; Valenza et al., 2014, 2015; Guo et al., 2015; Hernando et al., 2016; Rakshit et al., 2016; Verkuil et al., 2016; Goessl et al., 2017; Lischke et al., 2018; May et al., 2018; Van Boxtel et al., 2018). As the use of ECG increases in ambulatory environments, removing motion artifacts has become more important to properly detect the QRS complex. Weak motion artifacts can lead to a slight wandering baseline in the electrocardiogram, and a QRS complex can be properly detected through conventional filtering. However, severe motion artifacts have a very large amplitude and can thus saturate the electrocardiography amplifier or distort the ECG waveform significantly, resulting in a loss of QRS information. Thus, it is obvious that ectopic beats due to a false QRS detection will increase.

The simplest method to correct unwanted beats is to delete ectopic beats, but eliminating ectopic beats reduces the total number of IBIs, which results in errors in the HRV analysis. Researchers have previously recommended avoiding deletion in artifact correction for HR variability spectrum analyses (Salo et al., 2001; Mateo and Laguna, 2003). Salo et al. found that errors in the HF and LF components of the short-term RR interval time series were more than 5% when less than 5% of the RR intervals had been deleted. These effects on the waveform also make deletion unsuitable for VLF and ULF component analyses (Salo et al., 2001). Furthermore, researchers have also reported that deletion editing may produce a false increase in α1 values in patients with acute myocardial infarction (Peltola et al., 2004) and in patients with coronary artery disease (Tarkiainen et al., 2007) in a detrended fluctuation analysis (DFA). These studies commonly indicate that removing IBIs is not recommended for a time-domain analysis of HRV and for a frequency domain analysis of HRV without interpolation. Therefore, various interpolation algorithms have been adapted and evaluated to calibrate the ectopic beating interval to prevent failure in the HRV analysis (Birkett et al., 1991; Lippman et al., 1994; Salo et al., 2001; Mateo and Laguna, 2003; Clifford and Tarassenko, 2005; Kim et al., 2007, 2009, 2012; Colak, 2009).

Interpolation replaces abnormal RR intervals with the interpolated RR interval, and unlike deletion-only, it removes the ectopic beat while maintaining the total number of IBIs. Interpolation is recommended for power spectral HRV analyses, especially when the RR interval time series contains ectopic beats or artifacts. The representative interpolation methods in the HRV analysis include zero-order, first order, spline, and non-linear predictive interpolation (Peltola, 2012). The zero-order interpolation method, which is referred to as nearest neighbor interpolation (NNI), replaces the abnormal RR interval with the previous RR interval. The first order interpolation method, which is referred to as linear interpolation (LI), derives a straight line connecting the adjacent RR intervals and calculates a new RR interval from the straight line. Cubic spline interpolation (CSI), which approximates a smooth curve through cubic polynomials fitted with multiple data values, is frequently used as spline interpolation. In addition, comparing–merging (Cheung, 1981), predictive autocorrelation (Albrecht and Cohen, 1998), non-linear predictive interpolation (Lippman et al., 1993), excluding RR interval segments with ectopic duration (Rottman et al., 1990; Lombardi et al., 1996), integral pulse frequency model (IPFM) (Mateo and Laguna, 2003; Solem et al., 2006), and non-linear filtering through wavelet-based trend removal (Thuraisingham, 2006) have also been proposed.

Many preexisting beat correction techniques have improved the significance of an HRV analysis. However, the quantitative relationship between the quantity of ectopic beats and the significance of HRV has not yet been investigated in depth. Previous studies analyzing the effects of ectopic beats examined the effects of missing beats in the time domain (Kim et al., 2007), frequency domain (Kim et al., 2009), and non-linear (Kim et al., 2012) HRV analyses. In those studies, the quantity of ectopic beats was defined based on the time duration, including the artifact, and they investigated how the HRV varied with the length of time including the noise. Those studies analyzed the significance of HRV according to the duration of noise and provided practical guidance. However, their research does not provide a strict quantitative evaluation because a capacitive sensor was used instead of a standard electrode to measure the ECG or the ECG used as reference cannot confirm whether or not it contains ectopic beats.

In summary, the specific heartbeat has already been confirmed to cause errors in HRV analyses, but the extent to which an ectopic beat is acceptable has not been quantitatively determined. For example, there is no evidence yet to quantitatively address the following problem: what is the number or ratio of the ectopic beat that could change the results of the HRV analysis? How many ectopic beats are allowed to produce a flawless HRV analysis? Thus, the purpose of our study is to quantitatively analyze how the quantity of ectopic heartbeats affects the HRV analysis.

This study also includes a quantitative review of the performance of ectopic beat correction algorithms according to the number of ectopic beats included in the electrocardiogram. A quantitative analysis of the effect of applying ectopic beat correction algorithms according to the number of ectopic beats could be applied in the development and application of ECG-based analytic methods that take ectopic beats into account in the future. Specifically, it is expected to be useful in studying the significance of heart rate variability measured during HRV calculations in a mobile environment containing motion noise.

## Materials and methods

### Data summary

This study complied with the Chonnam National University (Gwangju, Republic of Korea) research ethics regulations, and all subjects signed informed consent forms before the experiment. We obtained ECG measurements from 30 volunteers and used data from 28 subjects, except for data duplicated by operational mistakes or data distorted due to severe motion artifacts. Finally, a dataset from 28 subjects (20 males, 8 females, age 22.0 ± 2.9 years, height 170.2 ± 7.3 cm, and weight 70.5 ± 15.4 kg) was used for the analysis. All data were recorded for 20 min with a 10 kHz sampling frequency in the supine position with the Lead II configuration. The subjects were asked to be at rest without sleeping or producing motion noise during the signal acquisition. No participant reported any cardiovascular disease. Also, all participants were instructed to avoid caffeine, alcohol and smoking a day before and during the experiment since these could affect the autonomic nervous activity. MP150, ECG100C, and Acknowledge 4.0 (Biopac Systems Inc., Santa Barbara, CA, USA) were used for 16-bit analog and digital conversion, ECG amplification and data recording, respectively. From the recorded ECG, the normal QRS dataset (*DB*_nQRS_) was constructed for each subject. To ensure the integrity of the QRS locations, each QRS location was checked and qualified by several experts using an in-house Matlab GUI to manually correct the QRS location after automatic QRS screening based-on Pan-Tompkins QRS detection algorithm (Pan and Tompkins, 1985). To remove the pre-existing ectopic beats, IBIs that increased by more than 32.5% or decreased by less than 24.5% with respect to the previous interval were removed before artificial ectopic beat generation (Kamath and Fallen, 1995).

### Artificial ectopic beat generation

**Figure 1:**
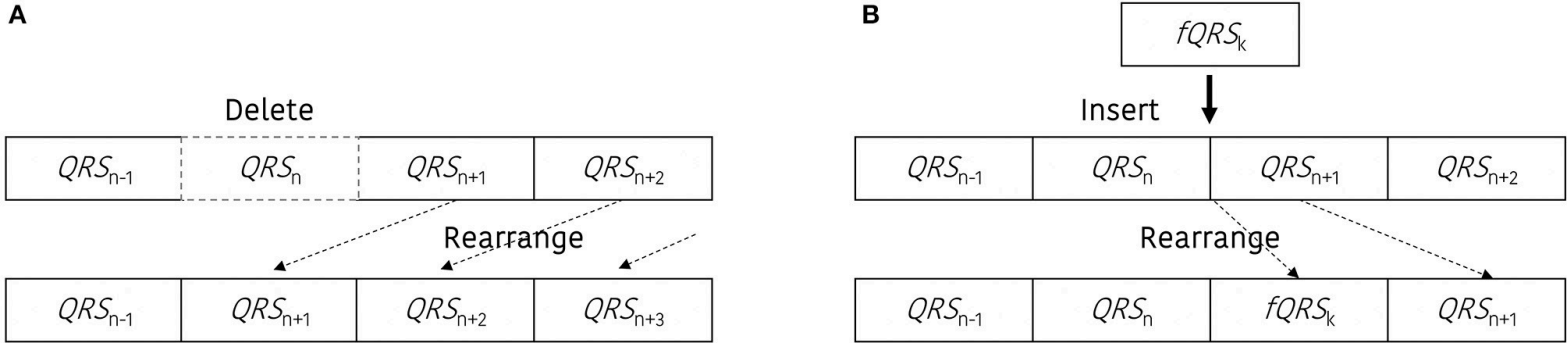
Graphical representation to update the dataset including the beat errors. **(A)** Procedure to generate the miss-detected QRS dataset, **(B)** procedure to generate the false-detected QRS dataset.

Artificial ectopic beats were generated with two types of error; miss detected QRS and false detected QRS. To construct the QRS dataset with ectopic beats (*DB*_eIBI_), we generate miss-detection by deleting the QRS location or create a false QRS by inserting additional QRS between two adjacent peaks. The total number of ectopic beats is determined with a predefined error rate, and the ratio between the number of miss detected QRS and the number of false detected QRS is randomly selected. Figure 1 shows a graphical representation of the beat error generation procedure. Figure 1A is a miss-detection generation procedure. The dashed box represents the miss-detected QRS that will be deleted, and the removed QRS location will be filled with the next adjacent QRS.

**Figure 2:**
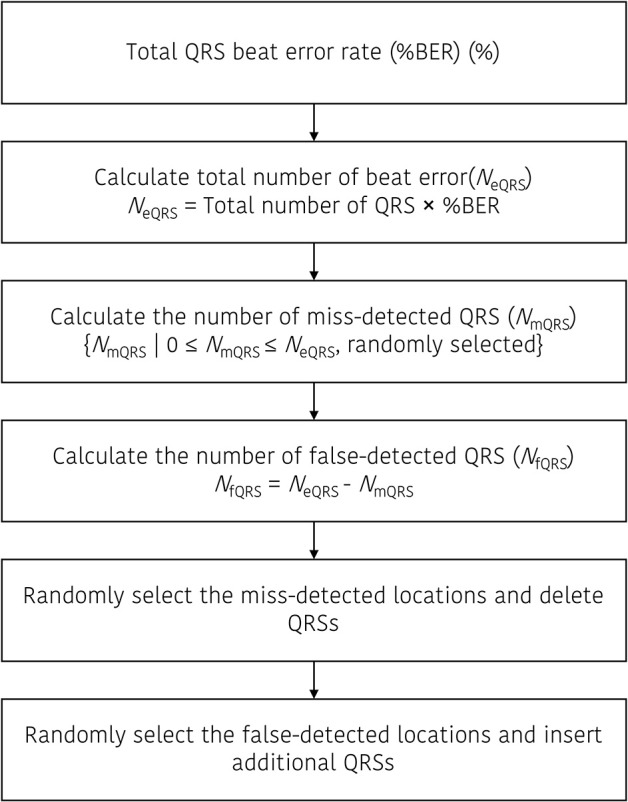
Procedure to update the QRS dataset that includes the beat errors.

Figure 1B presents an example of the false-detected QRS (*fQRS*) generation procedure. In this example, the *fQRS*_k_ refers to the *k*-th false detected QRS that is randomly selected between the *QRS*_n_ and *QRS*_n+1_. The updated QRS dataset that includes the false detected QRS location will be rearranged after the insertion of the *fQRS* between the adjacent QRS locations. The procedure to update the QRS dataset including the beat error is described in Figure 2.

### Ectopic restoration

To investigate the effect of the interpolation in the HRV analysis with ectopic beats, we applied the representative interpolation methods after thresholding based on the standard deviation. To create the restored IBI database (*DB*_rIBI_), we removed the ectopic IBI from the *DB*_eIBI_ and then filled the deleted value with interpolated data. In this procedure, we first remove the abnormal IBI. According to Kamath's suggestion, we considered an abnormal heartbeat when the heartbeat interval increased by more than 32.5% or decreased by more than 24.5% compared to the previous heartbeat interval (Kamath and Fallen, 1995). Equation (1) shows the mathematical expression for the thresholding method to acquire the ectopic-removed IBIs (*IBI*_ER_).

(1) $$\begin{array}{r} IBI_{\text{ER}}=\left\{IBI|\;0.675IBI_{n-1}<IBI_{n}<1.245IBI_{n-1}\right\} \end{array}$$

During interpolation, we used three types of general interpolation methods; including NNI, LI, and CSI. NNI is the simplest method to conduct multivariate interpolation in one or more dimensions. The nearest neighbor algorithm selects the value of the nearest point and does not consider the values of the neighboring points at all, yielding piecewise-constant interpolant. The LI is another simple method. In general, linear interpolation takes two data points, say (*x*_a_, *y*_a_) and (*x*_b_, *y*_b_), and the interpolant (*x, y*) is given by Equation (2).

(2) $$\begin{array}{r} y=\frac{x-x_{a}}{x_{b}-x_{a}}\left(y_{b}-y_{a}\right)+y_{a} \end{array}$$

The LI is quick and easy, but it is not very precise. Another disadvantage is that the interpolant is not differentiable at the point *x*_k_. Moreover, the error of the linear interpolation is proportional to the square of the distance between the data points. The error in some other methods, including polynomial interpolation and spline interpolation, is proportional to higher powers of the distance between the data points, and these methods also produce smoother interpolants.

The CSI uses three order polynomials in each of the intervals and chooses the polynomial pieces such that they fit smoothly together. The resulting function is called a spline. Like polynomial interpolation, spline interpolation incurs a smaller error than linear interpolation with smoother interpolant. However, the interpolant is easier to evaluate than the high-degree polynomials used in polynomial interpolation, and spline interpolation requires a higher computational load.

### HRV metrics

An HRV time domain analysis was performed with the different indices proposed by Taskforce in 1996 (Taskforce, 1996), including average NN interval (AVNN), standard deviation of NN interval (SDNN), standard deviation of successive difference between adjacent NN intervals (SDSD), root mean square of successive difference between adjacent NN intervals (RMSSD), the number of pairs of successive NNs that differ by more than 50 ms (NN50), and proportion of NN50 in total NN intervals (pNN50). The variables for long-term HRV monitoring, such as the mean of the 5-min standard deviation of the NN interval (SDNN index) and standard deviation of average NN intervals (SDANN) were excluded in this study as we focused on short-term physiological activities.

For the HRV frequency domain analysis, the interbeat intervals were transformed to an evenly-sampled time series by resampling at a 4 Hz sampling frequency after cubic spline interpolation with 1 kHz interpolation frequency, and then both the mean and linear trends were removed. The power spectral density was estimated by the fast Fourier transform (FFT), non-parametric spectral analysis method. To assess the HRV in the frequency domain, we measured and assembled the values of the very low frequency (VLF, 0.0033 to 0.04 Hz), low frequency (LF, 0.04 to 0.15 Hz), high frequency (HF, 0.15 to 0.4 Hz), LF/HF ratio (LF/HF), normalized LF (nLF), and normalized HF (nHF).

A non-linear analysis is another effective method for HRV analysis with non-stationary and non-linear characteristics. In this research, we used a Poincaré plot to evaluate the dynamic automatic modulation and approximated entropy (ApEn) that describes the complexity or R-R behavior (Beckers et al., 2001; Yang, 2006). The Poincaré plot is quantified by measuring SD1 and SD2. SD1 indicates the standard deviation of the Poincaré plot perpendicular to the line-of-identity, while SD2 represents the standard deviation of the Poincaré plot along the line-of-identity. The HRV metrics used in the analysis are described in Table 1.

**Table 1:** HRV metrics used in this study.

**Domain**	**HRV metric**	**Unit**	**Description**
Time	AVNN	ms	Average normal-to-normal interval
	SDNN	ms	Standard deviation of normal-to-normal interval
	SDSD	ms	Standard deviation of successive difference of normal-to-normal interval
	RMSSD	ms	Root mean square of successive difference of normal-to-normal interval
	pNN50	%	Percentage of normal-to-normal interval exceeds 50 ms
Frequency	TP	ms^2^	Total frequency power
	VLF	ms^2^	Very low frequency power of HRV (≤ 0.04 Hz)
	LF	ms^2^	Low frequency power of HRV (0.04-0.15 Hz)
	HF	ms^2^	High frequency power of HRV (0.15-0.4 Hz)
	LF/HF	n.u.	Ratio LF/HF
	nLF	n.u.	LF power in normalized units
			LF/(Total Power–VLF)
	nHF	n.u.	HF power in normalized units
			HF/(Total Power–VLF)
Non-linear	SD1	ms	Standard deviation of points (*QRS*_n_, *QRS*_n+1_) perpendicular to the axis of line of identity
	SD2	ms	Standard deviation of points (*QRS*_n_, *QRS*_n+1_) along the axis of line of identity
	ApEn	n.u.	Approximate entropy or normal-to-normal interval

### Simulation protocol

**Figure 3:**
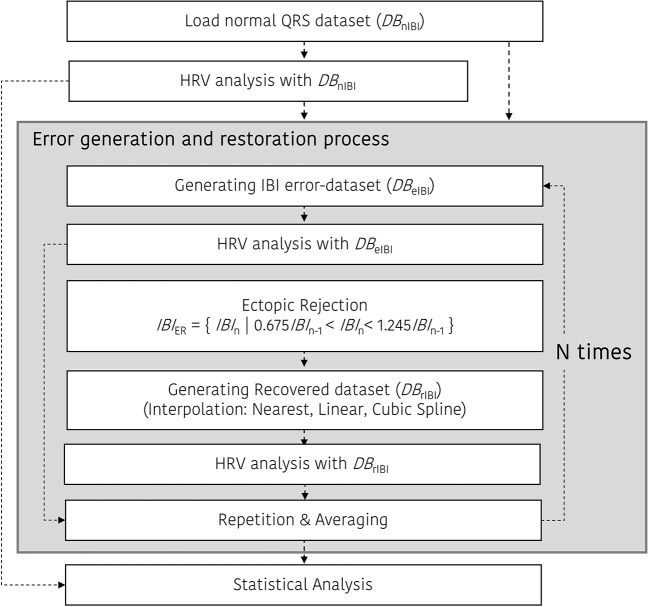
Ectopic-HRV simulation protocol.

Figure 3 shows the ectopic-HRV simulation protocol. There are three simulations for HRV analysis in the time and frequency domain: *DB*_nIBI_, *DB*_eIBI_, and *DB*_rIBI_. After that, ectopic IBI was corrected using a restoration procedure mentioned above. In the case of the beat error and restoration simulation, the beat error rate varied from 0 to 50% in 0.1% increments, and the simulation was repeated *N* times to obtain a more normalized result. In our simulation, the number of repetitions, *N*, is set to 30.

### Statistical analysis

After generating each of the simulation results, we calculated the percentage of fractional change (%FC) between the results of the HRV analyses derived from each dataset: *DB*_nIBI_, *DB*_eIBI_, and *DB*_rIBI_. Then, the statistical significance in the difference of *DB*_eIBI_ and *DB*_rIBI_ was compared to that with *DB*_nIBI_. The %FC is calculated by dividing the HRV variable derived from *DB*_eIBI_ or *DB*_rIBI_ with the HRV variable derived from *DB*_nIBI_ using Equation (3).

In the statistical validation, we check the data normality using the Kolmogorov-Smirnov test, and then performed a paired *t*-test when data normality was secured or used the Wilcoxon signed rank test when data normality was not achieved. All statistical analyses were conducted using IBM SPSS Statistics software, Version 23.0 (IBM Corp., Armonk, NY, USA).

(3) $$\begin{array}{r} \%FC=\frac{{Variable}_{Errororinterpolated}}{{Variable}_{normal}}\times 100 \end{array}$$

## Results

### Ectopic beat generation and interpolation

**Figure 4:**
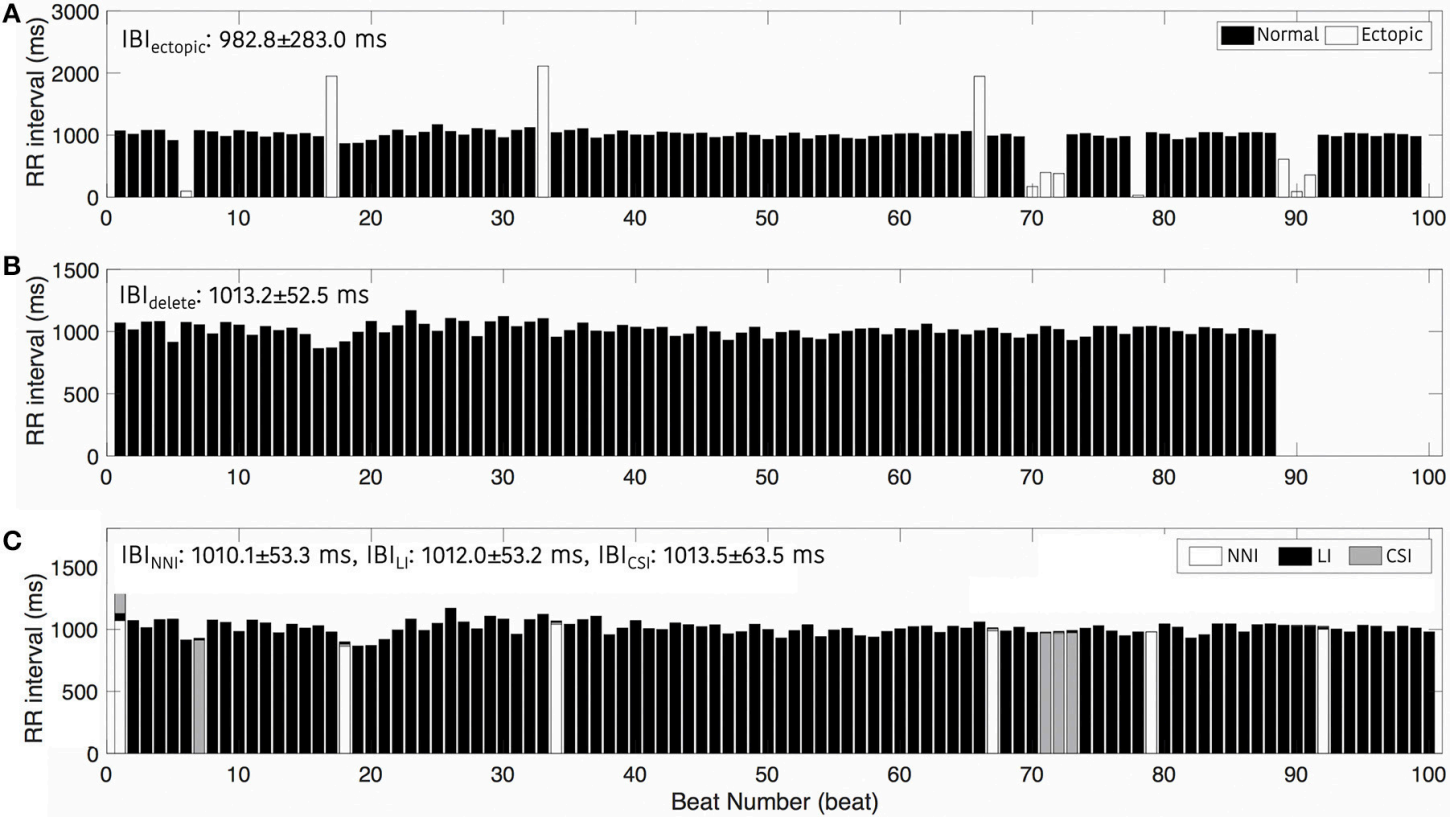
Interbeat intervals. **(A)** Including ectopic beats, **(B)** After removing ectopic beats, and **(C)** After interpolation. NNI, Nearest Neighbor Interpolation; LI, Linear Interpolation; CSI, Cubic Spline Interpolation.

Figure 4 shows an example of the RR intervals including ectopic events (Figure 4A), excluding ectopic by deleting (Figure 4B), and after interpolation (Figure 4C). This result shows a noticeable change in IBIs with an ectopic beat. In particular, we regard the rapid increase in the RR interval as a missing beat and a drastic decrease in the RR interval as a false detection beat. In Figure 4B, when the ectopic beat has been removed, the number of total beats decreases. Figure 4 also shows an example of IBI changes according to deletion or interpolations. IBI including ectopic (*IBI*_ectopic_) was 982.8 ± 283.0 ms, *IBI*_delete_, which simply removed ectopic, was 1013.8 ± 52.5 ms and IBI was 1010.1 ± 53.3 ms, 1012.0 ± 53.2 ms, and 1013.5 ± 63.5 ms after nearest neighbor (*IBI*_NNI_), linear (*IBI*_LI_), and cubic spline interpolation (*IBI*_CSI_), respectively.

### Fractional changes and statistical significance

**Figure 5:**
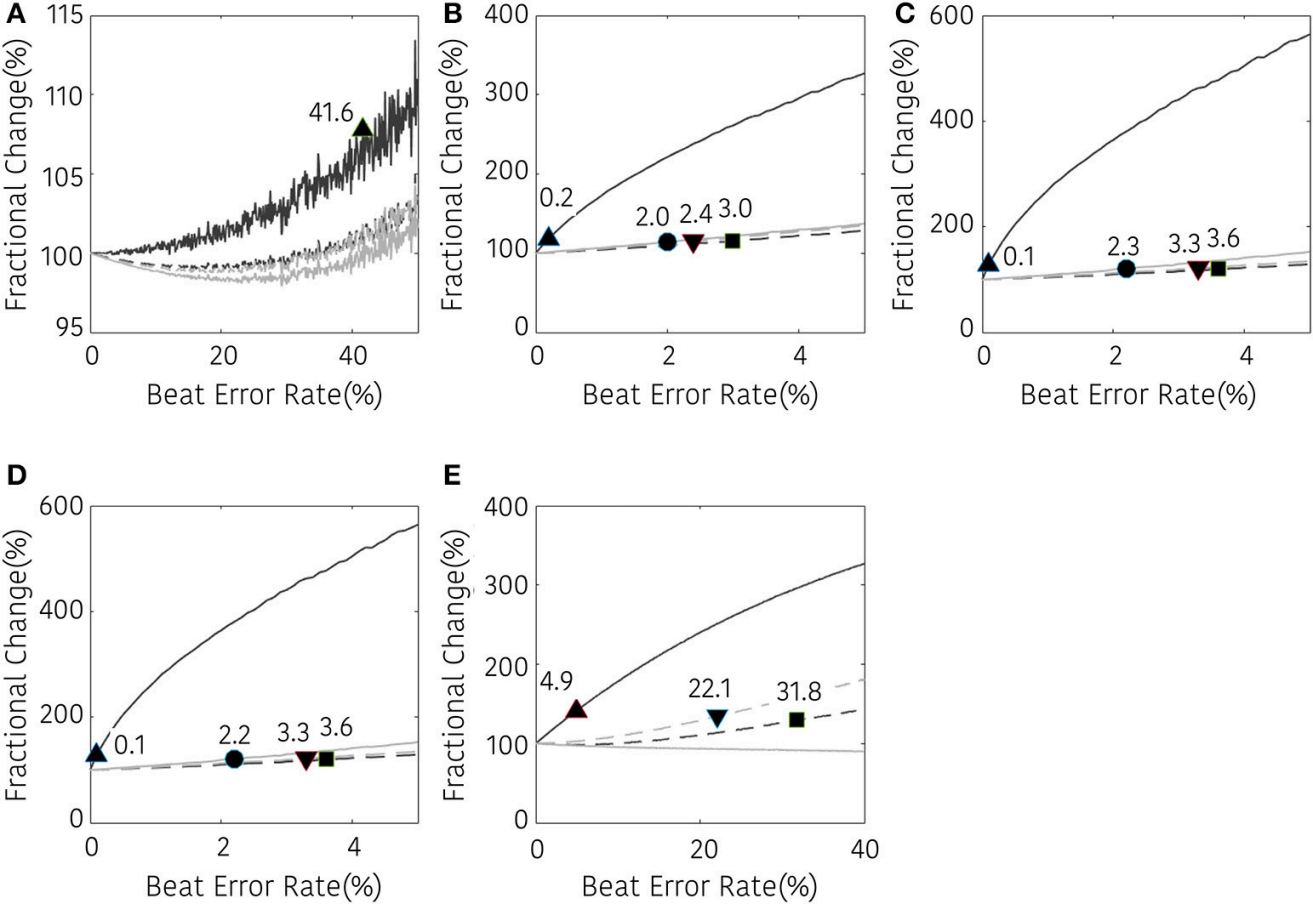
Result of time-domain HRV analysis according the beat error rate. The number in the figure represents the beat error rate that begins with a significant difference. The dark solid line, bright solid line, dark dashed line, and bright dashed line indicate non-interpolated, NNI, LI, and CSI, respectively. **(A)** AVNN, **(B)** SDNN, **(C)** SDSD, **(D)** RMSSD, **(E)** pNN50. Markers (▴, •, ■, ▾) represent the position of the error rates that start to have a statistically significant difference compared to the HRV variable derived with ectopic-free IBIs.

**Figure 6:**
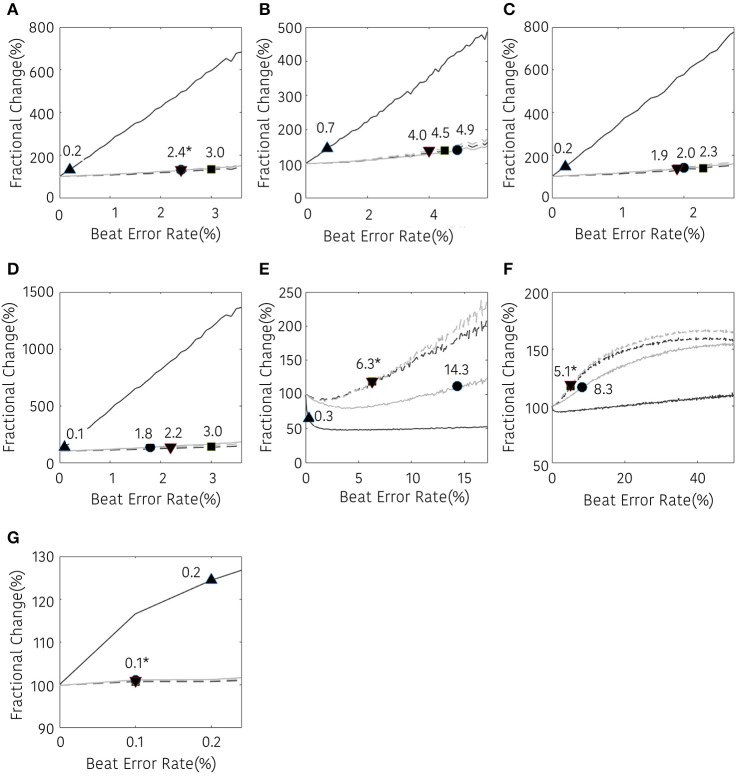
Result of frequency-domain HRV analysis according the beat error rate. The number in the figure represents the beat error rate that begins with a significant difference. The dark solid line, bright solid line, dark dashed line, bright dashed line indicate non-interpolated, NNI, LI, and CSI, respectively. **(A)** TP, **(B)** VLF, **(C)** LF, **(D)** HF, **(E)** LF/HF, **(F)** nLF, **(G)** nHF. Markers (▴, •, ■, ▾) represent the position of the error rates that start to have a statistically significant difference compared to the HRV variable derived with ectopic-free IBIs.

**Figure 7:**
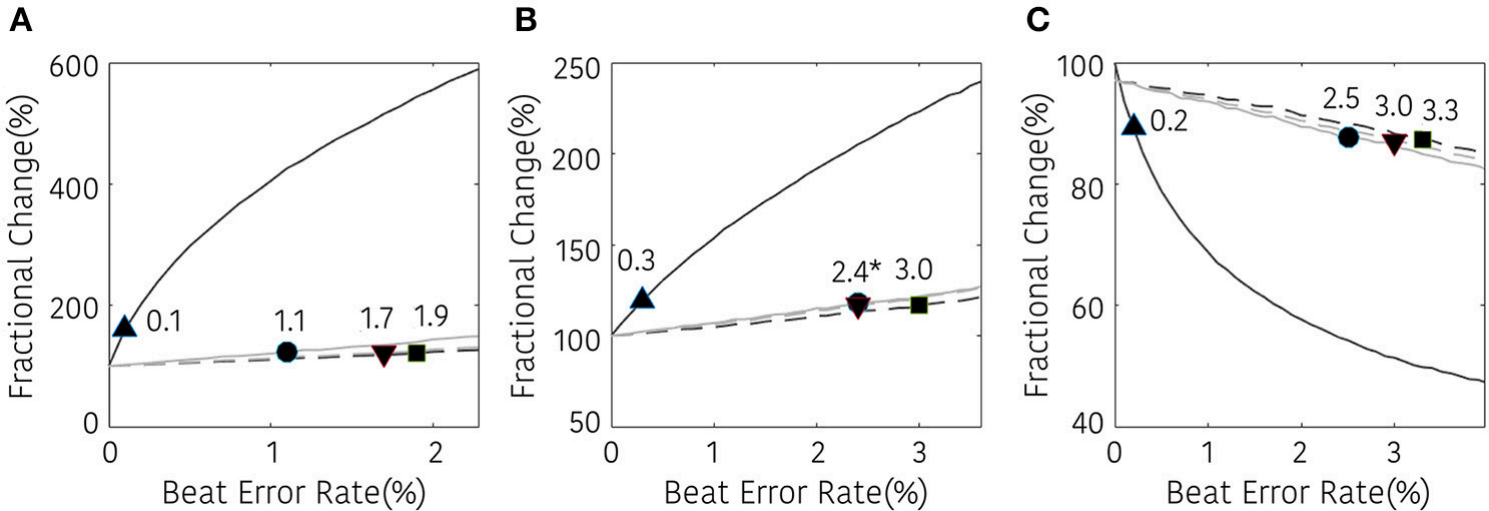
Result of non-linear HRV analysis according to the beat error rate. The number in the figure represents the beat error rate that begins a significant difference. The dark solid line, bright solid line, dark dashed line, bright dashed line indicate non-interpolated, NNI, LI, and CSI, respectively. **(A)** SD1, **(B)** SD2, **(C)** ApEn (▴, •,■, ▾) represent the position of error rates that start to have a statistically significant difference compared to the HRV variable derived with ectopic-free IBIs.

Figures 5–7 represents the fractional changes in the HRV variables depending on the beat error rate in the time-domain analysis, frequency-domain analysis, and non-linear analysis. The numbers in the figure represent the error rates that start to have a statistically significant difference compared to the HRV variable derived with ectopic-free IBIs. Prior to the statistical validation, data normality was tested using the Kolmogorov-Smirnov test, then the statistical differences were assessed using a Wilcoxon rank-sum test or a paired *t*-test according to data normality. For example, in Figure 5A, 41.6 means that AVNN will have a statistical difference from the ectopic-free HRV at a 41.6% beat error rate when no interpolation is performed (black solid line). In the time domain analysis, AVNN preserves its statistical significance even at 50% beat error when interpolated. However, when the interpolation was not performed, there was a statistically significant difference from the beat error of 41.6%. In the case of SDNN, 0.2% of beat error was found to be statistically significant when interpolation was not performed, and 2.0, 3.0, and 2.4% of beat error were allowed for NNI, LI, and CSI, respectively. SDSD, and RMSSD showed the same characteristics. Significant statistical differences were found at 0.1% of the beat error when interpolation was not performed in both variables. After interpolation, error tolerance extended to 2.2, 3.6, and 3.3% according to NNI, LI, and CSI, respectively. The pNN50 was relatively robust against beat errors. pNN50 showed no statistical difference up to 4.9% of the beat error without interpolation, 22.1% of beat error with spline interpolation, and 31.8% of beat error with first order interpolation. Even with NNI, there was no statistical difference in 50% of beat error.

The results of the frequency domain analysis of HRV are shown in Figure 6. Most results of the frequency analysis showed a similar pattern. In the case with no interpolation, a statistical difference was observed at a very low beat error rate, and the error tolerance increased with interpolation. The beat error rate, which starts to show a significant difference when not interpolating, is 0.2, 0.7, 0.2, 0.1, 0.3, and 0.2% in TP, VLF, LF, HF, LF/HF, and nHF, respectively. The maximum beat error rate according to the interpolation method was different for each HRV variable. For the TP, the beat error rate was 2.4, 3.0, and 2.4% for the NNI, LI, and CSI, respectively, for statistically significant results. The allowable beat error rate was expanded to 4.9, 4.5, 4.0% for VLF, 2.0, 2.3, 1.9% for LF 1.8, 3.0, 2.2% for HF, 14.2, 6.3, and 6.3% for LF/HF, 8.3, 5.1, and 5.1% for nLF and 0.1, 0.1, and 0.1% for nHF, respectively, when the NNI, LI and CSI were applied. Strangely, nLF did not show any significant difference from the original result in 50% beat error without interpolation, but it showed a significant difference from the original signal when interpolation was performed.

The non-linear analysis showed a similar tendency according to the interpolation method. SD1, SD2, and ApEn were significantly different at 0.1, 0.3, and 0.2% beat error rates, respectively, when interpolation was not performed. The range of the beat error rate which did not show a significant difference was expanded to 1.1, 1.9, and 1.7% for SD1, 3.0, 2.4, and 2.4% for SD2, and 2.5, 3.3, and 3.0% for ApEn, when applying the NNI, LI, and the CSI, respectively. Figure 7 shows the fractional change and the beginning of the significant difference of the HRV with non-linear analysis according to the beat error rate.

### Correlation analysis

The correlation coefficient analysis shows a decrease in the correlation coefficient as the beat error rate increases. Table 2 shows the correlation coefficient at a beat error rate when a significant difference is observed compared to ectopic-free HRV based on statistical validation (In parentheses, the beat error rate at which the significant difference begins). In most results of the correlation coefficient analysis, a strong correlation (>0.9) was observed. Especially, in the time domain, every variable showed a high correlation. In the frequency domain, normalized variables such as LF/HF, nLF, and nHF showed slightly lower correlation. In the case of LF/HF, the beat error rate with statistical error was relatively high, but the correlation was noticeably low. In the case of nLF and nHF, LI, and CSI also showed significant results with high beat error rate, but a low correlation was observed. In the non-linear analysis, the results showed a high correlation in all variables and relatively low correlation in ApEn. The interesting fact is that the beat error rate, which starts to show a significant difference when not interpolating and when interpolating, shows a large difference, but there is no large difference in the correlation. In some cases, the correlation is higher when the interpolation is not performed. This means that the correlations do not guarantee statistical significance in the HRV analysis including the ectopic beat, suggesting that the number of subjects is insufficient.

**Table 2:** Correlation coefficient of HRV metrics by the interpolation method.

**Domain**	**HRV metric**	**Non-interpolated**	**Interpolation method**		
			**NNI**	**LI**	**CSI**
Time	AVNN	0.920 (41.6%)	–	–	–
	SDNN	0.994 (0.2%)	0.997 (2.0%)	0.993 (3.0%)	0.993 (2.4%)
	SDSD	0.986 (0.1%)	0.998 (2.2%)	0.983 (3.6%)	0.994 (3.3%)
	RMSSD	0.986 (0.1%)	0.998 (2.2%)	0.983 (3.6%)	0.994 (3.3%)
	pNN50	0.999 (4.9%)	–	0.986 (31.8%)	0.992 (22.1%)
Frequency	TP	0.992 (0.2%)	0.993 (2.4%)	0.981 (3.0%)	0.991 (2.4%)
	VLF	0.971 (0.7%)	0.948 (4.9%)	0.988 (4.5%)	0.974 (4.0%)
	LF	0.985 (0.2%)	0.978 (2.0%)	0.968 (2.3%)	0.985 (1.9%)
	HF	0.995 (0.1%)	0.998 (1.8%)	0.993 (3.0%)	0.997 (2.2%)
	LF/HF	0.837 (0.3%)	0.234 (14.3%)	0.652 (6.3%)	0.604 (6.3%)
	nLF	–	0.786 (8.3%)	0.905 (5.1%)	0.898 (5.1%)
	nHF	0.887 (0.2%)	0.995 (0.1%)	0.991 (0.1%)	0.993 (0.1%)
Non-linear	SD1	0.932 (0.1%)	0.998 (1.1%)	0.986 (1.9%)	0.995 (1.7%)
	SD2	0.994 (0.3%)	0.997 (2.4%)	0.981 (3.0%)	0.995 (2.4%)
	ApEn	0.889 (0.2%)	0.872 (2.5%)	0.838 (3.3%)	0.847 (3.0%)

*NNI, Nearest Neighbor Interpolation; LI, Linear Interpolation; CSI, Cubic Spline Interpolation*.

## Discussion

### Suggestion for non-ectopic HRV

Our result suggests that the results of the HRV analysis could be distorted by just a few ectopic beats. Most of the HRV variables showed a significant difference despite just *0.x*% of ectopic beats being included. Interpolation could be a good complement. Although there are differences depending on the variables, interpolation expanded the significance range by as little as 1–2% and as large as 10%. In terms of securing significance, interpolation may be a good method, but there is still a problem of estimating the value as too large or too small. In most variables, such as SDNN, SDSD, RMSSD, pNN50, TP, VLF, LF, HF, nLF, SD1, and SD2, the values were overestimated after interpolation. On the other hand, underestimation was observed in ApEn, and AVNN, while LF/HF and nHF showed an unspecified variation.

### Comparison of interpolation methods

In most cases, the use of interpolation reduced the error caused by ectopic beats, and the reduction in error varies according to the particular method that is used. Among the three interpolation methods used in this study, the zero-order and first-order interpolation showed similar trends, but spline interpolation showed a slight difference. The interpolation method exhibited a difference in mitigating the ectopic error. Overall, LI showed the best performance. In the interpolation method, LI and CSI showed a better performance in the time-domain and high-frequency-related variables, whereas NNI showed better results in the interpolation of low-frequency components such as VLF, LF, LF/HF, and nLF. Linear interpolation and spline interpolation can be assumed to interpolate an increasing or decreasing trend as it is, and the power can be estimated. The interpretation of the above results is consistent with the results presented by Salo et al. (2001).

## Conclusion

This study made quantitative observations of the changes in HRV results for errors in beat detection. This study quantifies the ectopic beat rate and observes the results of the HRV analysis, unlike in previous studies that compared the HRV result with the length of the bit detection error interval or comparing before / after ectopic elimination.

The findings of this study are somewhat surprising. Although the ratio of ectopic beats in most HRV variables was very low at 0.*x*%, a statistical difference was shown. In the case in which interpolation is applied, a statistically significant difference occurred when the ectopic beat rate exceeded several percent. This result suggests that the ectopic beat should be removed in the HRV analysis and also provides a perspective of the accuracy requirements of electrocardiogram QRS detection algorithms for HRV analysis. The results show that even if interpolation is considered, a QRS detection accuracy of about 97% or more is required to obtain a statistically significant HRV result. However, this study has limitations in that the experimental conditions are limited to the resting condition for healthy adult participants. Since the HRV may be affected by age, health status, and exercise status, expansion to a more diverse and larger population of subjects is required to establish a more general outcome.

## Author contributions

HS planned and envisioned this study. HS and AC were experimentally designed and simulated. HS and AC have prepared and reviewed the manuscript.

### Conflict of interest statement

The authors declare that the research was conducted in the absence of any commercial or financial relationships that could be construed as a potential conflict of interest.
